# Where should siRNAs go: applicable organs for siRNA drugs

**DOI:** 10.1038/s12276-023-00998-y

**Published:** 2023-07-10

**Authors:** Insook Ahn, Chanhee S. Kang, Jinju Han

**Affiliations:** 1 Graduate School of Medical Science and Engineering, Korea Advanced Institute for Science and Technology (KAIST), Daejeon, Republic of Korea; 2 BioMedical Research Center, KAIST, Daejeon, Republic of Korea

**Keywords:** RNAi therapy, RNAi

## Abstract

RNA interference mediated by small interfering RNAs (siRNAs) has been exploited for the development of therapeutics. siRNAs can be a powerful therapeutic tool because the working mechanisms of siRNAs are straightforward. siRNAs determine targets based on their sequence and specifically regulate the gene expression of the target gene. However, efficient delivery of siRNAs to the target organ has long been an issue that needs to be solved. Tremendous efforts regarding siRNA delivery have led to significant progress in siRNA drug development, and from 2018 to 2022, a total of five siRNA drugs were approved for the treatment of patients. Although all FDA-approved siRNA drugs target the hepatocytes of the liver, siRNA-based drugs targeting different organs are in clinical trials. In this review, we introduce siRNA drugs in the market and siRNA drug candidates in clinical trials that target cells in multiple organs. The liver, eye, and skin are the preferred organs targeted by siRNAs. Three or more siRNA drug candidates are in phase 2 or 3 clinical trials to suppress gene expression in these preferred organs. On the other hand, the lungs, kidneys, and brain are challenging organs with relatively few clinical trials. We discuss the characteristics of each organ related to the advantages and disadvantages of siRNA drug targeting and strategies to overcome the barriers in delivering siRNAs based on organ-specific siRNA drugs that have progressed to clinical trials.

## Drug development: Refining RNA-based therapeutics to target multiple organs

Refining the design and delivery of RNA-based drugs could improve the chances of targeting diseases in complex organs. Gene expression is regulated by small interfering RNAs (siRNAs), which bind to messenger RNA sequences, preventing subsequent gene expression. Drugs based on siRNA show promise as safe and specialised for multiple diseases. Jinju Han and co-workers at the Korea Advanced Institute for Science and Technology in Daejeon, South Korea, reviewed the current status of siRNA drugs. Five siRNAs approved by the US Food and Drug Administration target the liver, while drugs targeting eye conditions and wound healing in the skin are in clinical trials. Targeting complex organs like the brain and lungs remains challenging, because the size of siRNAs and their delivery mechanisms must be adjusted in order to pass safely into target regions.

## Introduction

Antisense transcripts to perturb the expression of target mRNAs have been widely used for genetic analyses. DNA plasmids expressing antisense transcripts of target mRNAs have been introduced into mammalian cells^1^, and antisense transcripts synthesized in vitro have been injected into frog oocytes^2,3^. The strategy of suppressing gene expression using antisense transcripts has also worked well in *C. elegans*. However, it was revealed that double-stranded RNAs (dsRNAs) are more potent in suppressing target genes than antisense transcripts^4^. Exogenous long dsRNAs in worms are processed into short RNA duplexes of ~21-22 nt by RNase III and loaded onto ARGONAUTE proteins to suppress target gene expression. This biological process of RNA interference (RNAi) is well conserved in diverse organisms, including humans.

**Fig. 1:**
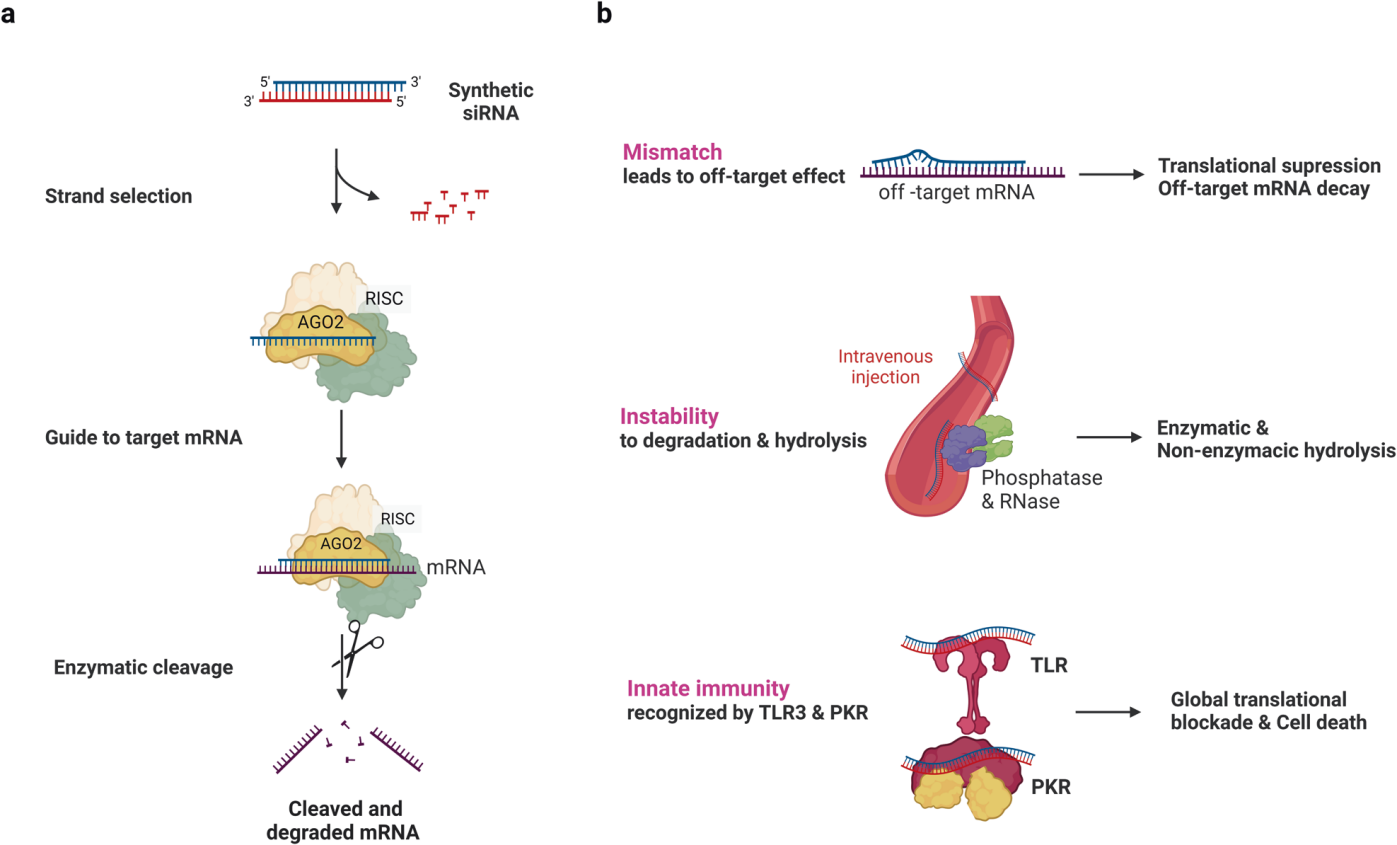
siRNA working mechanism and its features. **a** General mechanism of action of synthetic small interfering RNA (siRNA). Exogenous RNA duplexes of ~21 nt are incorporated into an RNA-induced silencing complex (RISC), which includes ARGONAUTE2 (AGO2). AGO2 cleaves the passenger strand and liberates the guide strand of the siRNA. The guide strand then guides the RISC to the target mRNA and leads to the cleavage of mRNA. **b** Considerations that point to the development of siRNAs as drugs: off-target effects caused by binding of the guide strand to nontarget mRNA with mismatches, RNA stability decreased by degradation and hydrolysis, and immune responses induced by dsRNA recognition.

To apply RNAi to human cells, a short form of RNA duplexes should be delivered because long dsRNAs induce innate immune responses^5^. Exogenous ~21 nt RNA duplexes are incorporated into AGO proteins, and one of the strands is selected as a guide strand depending on the thermodynamic stability at the 5’ end of the RNA duplexes^6^. When the guide strand of siRNA forms a complex with AGO2 and binds to target RNAs with complementary sequences without mismatches, the ribonucleoprotein complex can cleave target RNAs **(**Fig. 1a**)**. AGO2, among the four AGO proteins in humans, possesses endonuclease activity that cuts the phosphodiester bond of the target RNAs located between the 10th and 11th nucleotides from the 5’ end of the guide strand^7^.

siRNAs have long been considered promising drug platforms because their working mechanisms have been well demonstrated, and siRNAs can be designed to target a specific RNA based on target sequences. Nevertheless, there are some factors to consider when developing siRNAs as drugs (Fig. 1b). siRNAs can bind to off-target RNAs with mismatches, which can result in translational suppression or decay of the off-target RNAs^8^. The off-targeting of siRNAs can be minimized by formulating siRNA sequences with computational algorithms^9^. The other issues that have arisen in using siRNAs as drugs are instability and potential immune reactions. RNAs are unstable and easily degraded by enzymatic and nonenzymatic hydrolysis. Unmodified naked siRNAs delivered into organisms through intravenous injection can be rapidly degraded because of vulnerability to RNases and phosphatases^10^. Moreover, a high concentration of unformulated and unmodified siRNAs that can be recognized by Toll-like receptor 3 (TLR3) or serine/threonine protein kinase (PKR)^11–14^ activate innate immune responses, leading to global translational blockade and cell death. To overcome these issues, the backbone and nucleotides of siRNAs are chemically modified^15^. Lastly, the delivery of negatively charged bulky siRNA to cells was improved by fusing molecules that can penetrate the lipid bilayers of cells or by encapsulating siRNAs into liposomes or lipid nanoparticles (LNPs). As a result of these tremendous efforts, the first siRNA-based drug, Patisiran, was approved by the United States Food and Drug Administration (US FDA) 20 years after the discovery of RNAi.

The approval of siRNA drugs has expanded the platforms used for oligonucleotide drug development. Before siRNA drugs were approved, only antisense oligonucleotide (ASO) drugs were used to control the expression of target genes that could not be targeted by traditional methods such as small molecules^16^. An ASO, a single-stranded oligonucleotide, binds to target RNAs and regulates gene expression in various ways. An ASO can perform RNase H1-mediated RNA cleavage, translational suppression, and splicing modulation^17^. The chemical modification of ASOs is indispensable for their stability and efficacy; however, the phosphorothioate or polyethylene glycol linkage backbone in ASOs can increase the binding affinity to unintended proteins, which is associated with toxicity^18–20^. siRNA drugs with less modified linkage backbones can be developed as alternatives to ASO drugs^21,22^.

As of December 2022, five siRNA drugs have been approved by the US FDA: Patisiran (Onpattro)^23^, Givosiran (Givlaari)^24^, Lumasiran (Oxlumo)^25^, Inclisiran (Leqvio)^26^, and Vutrisiran (Amvutta)^27^ **(**Table 1**)**. All five approved siRNA drugs target mRNAs expressed in the liver. This is not very surprising because siRNAs delivered into an animal rapidly accumulate in the liver, a major organ for detoxifying exogenous materials. However, siRNA drugs targeting mRNAs expressed in other organs are also under development. In this review, we discuss the features of organs from the perspective of siRNA targeting. We focus on a few organs that are targeted by currently available siRNA drugs and siRNAs in phase 2 and 3 clinical trials.

**Table 1:** siRNA drugs approved by the FDA as of 2022.

Drug/Trade name	Date of Approval	siRNA Carrier	Routes of administration	Indication and usage	Target organ	Target gene	Reference
Patisiran/Onpattro	August 10, 2018	Lipid nanoparticles	intravenous	Adult patients with hereditary transthyretin mediated (hATTR) amyloidosis	Liver	transthyretin (TTR)	^23^
Givosiran/Givlaari	November 20, 2019	GalNAc-conjugation	subcutaneous	Adult patients with acute hepatic porphyria (AHP)	Liver	aminolevulinate synthase 1 (ALAS1)	^24^
Lumasiran/Oxlumo	November 23, 2020	GalNAc-conjugation	subcutaneous	Adult and pediatric patients with primary hyperoxaluria type 1 (PH1)	Liver	hydroxy acid oxidase 1 (HAO1)	^25^
Inclisiran/Leqvio	December 21, 2021	GalNAc-conjugation	subcutaneous	Adult patients with heterozygous familial hypercholesterolemia or clinical atherosclerotic cardiovascular disease.	Liver	proprotein convertase subtilisin/kexin type 9 (PCSK9)	^26^
Vutrisiran/amvuttra	June 13, 2022	GalNAc-conjugation	subcutaneous	Adult patients with hereditary transthyretin mediated (hATTR) amyloidosis	Liver	transthyretin (TTR)	^27^

## Preferred organs targeted by siRNAs: Liver, eye and skin

Different strategies of siRNA delivery are being developed to target various organs precisely. Among the many organs, we categorized the liver, eye, and skin as preferred organs targeted by siRNAs. The first siRNA drug to enter clinical trials was AGN211745 (siRNA-027)^13^, targeting the eye. All siRNA drugs approved by the FDA target the liver^23–27^. Moreover, additional siRNA candidates targeting the liver, eye, and skin have progressed to phase 2 or 3 clinical trials. This section will discuss organs that are the preferred targets of siRNAs.

### Liver

The liver is an essential organ responsible for numerous functions, including protein synthesis, detoxification, and the production of necessary biochemicals for sustaining life. Most drugs are metabolized in the liver. In this process, enzymes located in the endoplasmic reticulum of liver cells convert lipid-soluble metabolites into water-soluble metabolites to excrete the metabolites originating from drugs through the kidneys^28^.

**Fig. 2:**
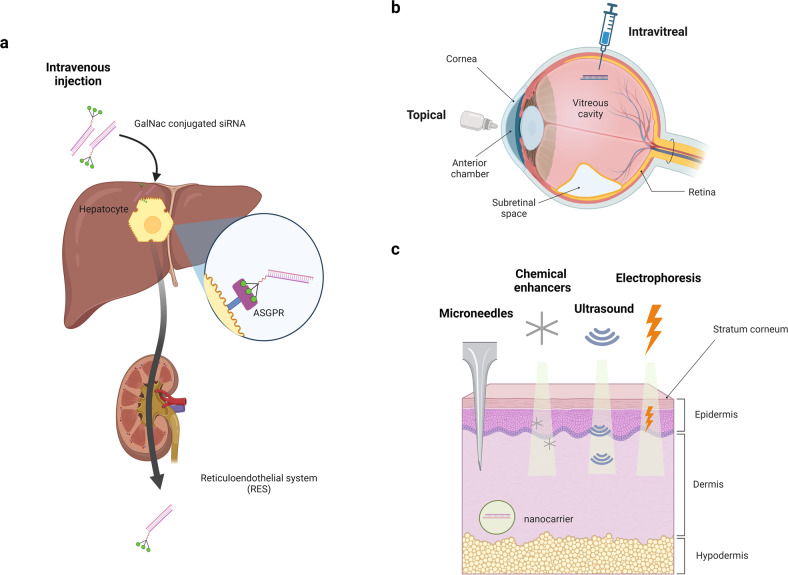
Preferred organs targeted by siRNAs: liver, eye and skin. The characteristics of the liver, eye, and skin as target organs of siRNA drugs are described. **a** siRNAs injected intravenously are accumulated in the liver through the reticuloendothelial system (RES). siRNAs conjugated with N-acetylgalactosamine (GalNAc) bind to hepatocyte-specific asialoglycoprotein receptor (ASGPR) with high affinity. **b** The eye is a clinically accessible organ with immune-privileged regions: the vitreous cavity, anterior chamber, and subretinal space. siRNA drugs can be administered topically or by intravitreal injections into the eye. **c** The skin is known to be the largest organ in the human body. Microneedles, chemical enhancers, ultrasound, electrophoresis, and nanocarrier delivery systems can be used to deliver siRNAs to the skin.

Both passive and targeted siRNA delivery can be used for the liver **(**Fig. 2a**)**. Passive delivery is determined by the intrinsic properties and anatomy of a specific tissue or cell type^29^. Recognition moieties or drug carriers are not necessary for passive delivery (also known as physiology-based targeting). The reticuloendothelial system (RES), a part of the immune system, preferentially captures vesicles and removes foreign bodies found in the blood circulation to protect the body from harmful effects. Therefore, siRNAs encapsulated by liposomes or LNPs tend to accumulate through passive delivery in the liver, spleen, lymph nodes, and kidneys, which are filtering organs belonging to the RES^30,31^. For targeted siRNA delivery, the asialoglycoprotein receptor (ASGPR) is utilized. The expression of ASGPR is negligible in other tissues but very high in parenchymal hepatocytes, which comprise 70~85% of the liver volume. siRNAs conjugated with N-acetylgalactosamine (GalNAc), a carbohydrate moiety, specifically bind to ASGPR with a high affinity that results in hepatocyte-specific uptake of the conjugates^32^. Four siRNA drugs among the five FDA-approved drugs, except for patisiran, are conjugated with GalNAc. GalNAc conjugation also provides additional stability to siRNAs (Table 1**)**.

Patisiran, the first FDA-approved siRNA drug, is delivered to the liver by LNPs to cure transthyretin-mediated amyloidosis (ATTR). ATTR is a fatal disease caused by the accumulation of misfolded TTR as amyloid fibrils in various tissues, including the heart, nerves, and gastrointestinal tract. While TTR proteins are expressed in all tissues of the human body, TTR mRNAs are primarily expressed in the liver. Vutrisiran, approved in 2022, also targets TTR mRNAs. Vutrisiran has advantages in the dosing method and interval. While patisiran is delivered by intravenous injection once every three weeks, vutrisiran is delivered subcutaneously only once every 3 months^27^. Givosiran was developed to treat acute hepatic porphyria (AHP), which is caused by the accumulation of neurotoxic intermediates called aminolevulinic acid (ALA) and porphobilinogen (PBG). ALA and PBG primarily accumulate in the liver and circulate through the body, resulting in neurological damage. Givosiran suppresses the expression of 5’-aminolevulinate synthase 1 (ALAS1), an enzyme required for ALA production, and reduces ALA and PBG levels. Lumasiran is used to treat primary hyperoxaluria type 1 (PH1). PH1 is caused by a deficiency in liver alanine glyoxylate-aminotransferase (AGT), which is responsible for the detoxification of glyoxylate. In the absence of AGT activity, glyoxylate is converted to oxalate, which forms insoluble calcium oxalate crystals in the kidney and other organs. Lumasiran cleaves hydroxy acid oxidase 1 (HAO1), an enzyme involved in oxalate synthesis^25^. Inclisiran was developed to treat heterozygous familial hypercholesterolemia and clinical atherosclerotic cardiovascular disease (ASCVD). Extremely high levels of low-density lipoprotein (LDL) and cholesterol (LDL-C) in plasma lead to premature ASCVD. Inclisiran delivered to the liver represses the synthesis of proprotein convertase subtilisin/kexin type 9 (PCSK9), a protein necessary for LDL-C metabolism^26^.

Moreover, fifteen siRNA therapeutics have progressed to phase 2 and 3 clinical trials (Table 2). The potential siRNA drugs in phase 3 include nedosiran (DCR-PHXC), ARO-APOC3, fitusiran (ALN-AT3SC), and revusiran (ALN-TTRSC), which target LDH, APOC3, SERPINC1, and TTR, respectively. Eleven siRNAs, cemdisiran, zilebesiran (ALN-AGT01), olpasiran (AMG 890), ARO-ANG 3, ARO-HBV (JNJ-3989), AB-729, SLN360, ARO-AAT, ARC-520 and ND-L02-s0201, are in phase 2. ARO-HBV, AB-729, and ARC-520 are for infectious diseases; fitusiran is for hematology diseases, and revusiran and ARO-AAT are for hereditary diseases. ND-L02-s0201 treats fibrotic diseases by inhibiting the expression of heat shock protein 47 (HSP47). All the other drugs are for metabolic diseases. Only ARC-520 and ND-L02-s0201 use a nanoparticle system to deliver siRNAs, while the rest of the drugs use the GalNAc conjugate delivery system.

**Table 2:** List of siRNA drug candidates in phase 2 and 3 clinical trials targeting the preferred organs: liver, eye, and skin.

Target organ	Drug name	Delivery system	Target gene	Disease	status
**Liver**	Nedosiran (DCR-PHXC)	GalNAc conjugate	LDH	Primary hyperoxaluria type 1,2	Phase 3, enrolling by invitation, NCT04042402
	Fitusiran (ALN-AT3SC)	GalNAc conjugate	SERPINC1	Hemophilia A and B	Phase 3, completed, NCT03549871
	ARO-APOC3	GalNAc conjugate	APOC3	Familial chylomicronemia syndrome	Phase 3, recruiting, NCT05089084
	Cemdisiran	GalNAc conjugate	C5	Paroxysmal nocturnal hemoglobinuria	Phase 2, active, not recruiting, NCT03841448
	Zilebesiran(ALN-AGT01)	GalNAc conjugate	AGT	Mild-to-moderate hypertension	Phase 2 (KARDIA-1), recruiting, NCT04936035
	Olpasiran(AMG 890)	GalNAc conjugate	LPA	Cardiovascular disease, patients with elevated serum lipoprotein A	Phase 2, active, not recruiting, NCT04270760
	ARO-HBV (JNJ-3989)	GalNAc conjugate	HBV RNAs	Hepatitis B	Phase 2, completed, NCT03365947
	AB-729	GalNAc conjugate	Viral antigens	Hepatitis B,D	Phase 2, active, not recruiting, NCT04820686
	ARO-ANG3	GalNAc conjugate	ANGPTL3	Mixed dyslipidemia	Phase 2, recruiting, NCT04832971
	Revusiran (ALN-TTRSC)	GalNAc conjugate	TTR	Transthyretin (TTR)-mediated amyloidosis	Phase 3, completed, NCT02319005
	SLN360	GalNAc conjugate	LPA	Cardiovascular diseases, atherosclerosis	Phase 2, Not yet recruiting, NCT05537571
	ALN-PCSSC	GalNAc conjugate	PCSK9	Homozygous familial hypercholesterolemia	Phase 2, completed, NCT02963311
	ARO-AAT	GalNAc conjugate	AAT	Alpha-1 antitrypsin deficiency	Phase 2, Active, not recruiting, NCT03946449
	ARC-520	Nanoparticle	HBV RNAs	Hepatitis B	Phase 2, terminated NCT02738008
	ND-L02-s0201	Nanoparticle	HSP47	Fibrosis	Phase 2, completed, NCT03538301
**Eye**	AGN211745 (sirna-027)	naked siRNA	VEGFR1 (FLT1)	Neovascular AMD	Phase 2, terminated, NCT00395057
	Bevasiranib	naked siRNA	VEGF	Neovascular AMD	Phase 3, Terminated, NCT00499590
	Tivanisiran (SYL1001)	naked siRNA	TRPV1	Dry eye disease with Sjogren syndrome	Phase 3, completed, NCT03108664
	Bamosiran(SYL040012)	naked siRNA	ADRB2	Elevated intraocular pressure	Phase 2, completed, NCT02250612
	Codosiran(QPI-1007)	naked siRNA	CASP2	Acute primary angle closure glaucoma	Phase 2a, completed, NCT01965106
	PF-0423655	naked siRNA	RTP801 (DDIT4)	Diabetic macular edema, choroidal neovascularization, diabetic	Phase 2, completed, NCT01445899
	RXI-109	naked siRNA	CTGF (CCN2)	Wet AMD	Phase 2, Unknown NCT02599064
	SYL1801	naked siRNA	NRARP	Wet AMD	Phase 2, recruiting, NCT05637255
**Skin**	Cotsiranib (STP705)	Nanoparticle	TGFB1 and COX-2 (PTGS2)	Hypertrophic scarring	Phase 2, recruiting, NCT04669808
	BMT101(cp-asiRNA)	naked siRNA	CTGF (CCN2)	Prevention of hypertrophic scarring	Phase 2a, recruiting, NCT04012099
	OLX10010	naked siRNA	CTGF (CCN2)	Reducing recurrence of hypertrophic scarring	Phase 2a, recruiting, NCT04877756
	RXI-109	naked siRNA	CTGF (CCN2)	Hypertrophic scarring	Phase 2, Completed NCT02030275

*Resource:* http://clinicaltrials.gov.

### Eye

The ocular system is located outside the cranium; thus, it is clinically accessible, unlike other central nervous system tissues. In particular, local delivery of siRNA to the ocular tissue is less complicated than delivery to other tissues. Local and near-direct delivery to the eye avoids the difficulties of systemic administration and minimizes systemic toxic effects. The additional advantage of delivering siRNA drugs to the eye is in the immune characteristics of the eye. The eye is an immune-privileged organ with limited local immune and inflammatory responses to maintain vision. This eye immune privilege is achieved through anatomical and biochemical mechanisms and is maintained in the vitreous cavity, anterior chamber, and subretinal space^33^. These features make the eye an excellent candidate for siRNA therapy. Indeed, the first siRNA drug that entered clinical trials was AGN211745 (sirna-027), targeting the eye.

Drugs are usually delivered to the eye directly by topical application (eye drops) or injection^34^ (Fig. 2b). For siRNA therapeutics, topical and intravitreal injection methods have likewise been used^35^. Topical administration is a noninvasive method and can be self-applied. However, topical administration is limited to diseases related to the anterior segment because there are multiple barriers to the back of the eye. Only approximately 10% of the applied dose is absorbed due to the physical barrier consisting of the corneal and conjunctival epithelium, nasolacrimal duct, and tears. Cell-penetrating peptide and silicon-based delivery approaches have been developed as siRNA delivery systems to increase the corneal permeability of drugs^36^. Various siRNAs using topical routes are under development^37,38^. The success of intravitreal injections of the anti-vascular endothelial growth factor (VEGF) “bevasiranib”^12^ has made the injection method common in drug delivery to the eye. Although piercing the retina can cause a physical break in blood tissue barriers and increase the risk of a systemic immune response^39,40^, injection of drugs into the vitreous humor is the most efficient method to deliver drugs to the posterior segments of the eye because it can bypass the natural barriers of the eyes. By using this advantage, siRNA drugs based on intravitreal injection are under development to treat diseases occurring in the posterior segment, such as age-related macular degeneration (AMD) and diabetic retinopathy (DR)^13,41^.

Currently, among siRNAs targeting the eye, eight drugs have progressed to phase 2 and 3 clinical stages: AGN211745 (sirna-027), bevasiranib, tivanisiran (SYL1001), bamosiran (SYL040012), codosiran (QPI-1007), PF-0423655, RXI-109, and SYL1801 (Table 2). Both AGN211745 (sirna-027) and bevasiranib have been developed to inhibit the VEGF signaling pathway for treating neovascular AMD. AGN211745 (sirna-027) targets VEGF receptor 1 (VEGFR1/FLT1), while bevasiranib targets VEGF itself. Tivanisiran (SYL1001) was developed to reduce ocular pain/discomfort in patients with dry eye disease by targeting Transient Receptor Potential Vanilloid 1. (TRPV1), a cation channel that acts as a nociceptive transducer. Bamosiran (SYL040012) targets the β2-Adrenergic Receptor (ADRB2) to reduce aqueous humor production and lower elevated intraocular pressure. Codosiran (QPI-1007) inhibits the loss of retinal ganglion cells (RGCs) and prevents optic neuropathy by targeting Caspase-2 (CASP2), which is highly expressed in RGCs during optical injury. PF-0423655 was developed for AMD patients by targeting RTP801 (DDIT4), a hypoxia-inducible gene overexpressed in choroidal neovascularization and diabetic retinopathy. SYL1801 targets NOTCH Regulated Ankyrin Repeat Protein (NRARP) and reduces the effects of the VEGF signaling pathway. Tivanisiran, bamosiran, and SYL1801 are administered topically, whereas AGN211745, bevasiranib, codosiran (QPI-1007), and PF-0423655 are administered by intravitreal injection.

### Skin

Human skin consists of three main layers, the epidermis, dermis, and hypodermis^42^, and it prevents excessive transepidermal water loss and protects the human body from the external environment, such as ultraviolet rays (UV), and from the entry of xenobiotics and microbes. The skin is the largest and most accessible organ in our body. In addition, it is relatively easy to apply treatment to local areas, monitor modified areas, perform tissue biopsies, and remove abnormal areas surgically for the skin. Thus, the skin is an attractive organ for the development of therapeutics.

Topical administration is a noninvasive drug delivery method commonly used for skin, but it is difficult to bypass a barrier called the stratum corneum (SC), which is the outer layer of the epidermis^43,44^. For transdermal drug delivery, physical methods including microneedles, chemical enhancers, ultrasound, electroporation, and iontophoresis have been developed^43^ (Fig. 2c). In addition to these general drug administration routes, delivering siRNA drugs by another approach using nanocarriers is being explored because nanocarriers have the advantages of biocompatibility, biodegradability, and versatility^45,46^.

Currently, among the siRNA studies that target the skin, four drugs, STP705 (cotsiranib), BMT101 (cp-asiRNA), OLX10010, and RXI-109, have progressed to clinical trials. The goal of these siRNA drugs is the same, treating hypertrophic scars caused by the excessive production of collagen from myofibroblasts during wound healing. All these siRNAs are delivered to the skin by intradermal injection. BMT101 (cp-asiRNA), OLX10010, and RXI-109 target connective tissue growth factor (CTGF), which is involved in the formation of hypertrophic scars and keloids. STP705 targets TGFβ1 and Cyclooxygenase-2 (COX-2/PTGS2), which modulate signaling pathways related to hypertrophic scars^47^.

#### Challenging organs to deliver siRNAs: the lungs, kidneys and brain

siRNA targeting the lung, kidney, and brain has rarely progressed to the clinical stage because there are many barriers to targeting the lung, kidney, and brain. This section will discuss organs that are challenging to target with siRNAs.

### Lungs

The main function of the lungs is the process of gas exchange called respiration. The lung is divided into a conducting region responsible for air conductance and a respiratory region where gaseous exchange takes place. The conducting region contains the nasal cavity, pharynx, trachea, bronchi, and bronchioles, while the respiratory region contains the respiratory bronchioles and alveoli. To deliver drugs more efficiently, intratracheal, intranasal, and inhalational drug delivery methods can be applied rather than systemic drug delivery methods. Drug loss is low by avoiding first-pass metabolism, and siRNA stability is better maintained because the airway contains fewer nucleases than the serum.

**Fig. 3:**
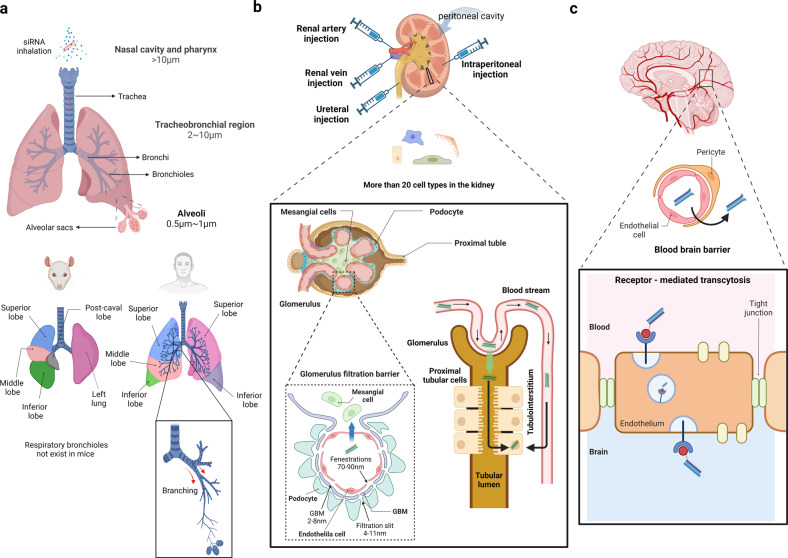
Challenging organs to deliver siRNAs: lungs, kidneys, and brain. Characteristics of the pulmonary system, kidneys, and brain as targeting organs of siRNA drugs are described. The lungs, kidneys, and brain have complex structures that limit a drug’s size. **a** siRNA is usually delivered to the lungs through inhalation, and the location of the drug distribution differs depending on the size of the drug. Mouse and human respiratory systems are very different in terms of anatomy. The mouse lung consists of four right lobes and one left lobe, whereas human lungs consist of three right and two left lobes. Respiratory bronchioles do not exist in mice, whereas humans have many bronchioles branched from bronchi. **b** The glomerular filtration barrier exists in the glomerulus, one of the components of the nephron. siRNAs can be delivered to proximal tubular cells from the apical or basolateral side. siRNAs can be directly delivered to the kidney through the renal artery, renal vein, ureteral, or intraperitoneal injection. **c** The brain has the most exceptional barrier, the blood‒brain barrier (BBB). Most siRNA nanoparticles cross the BBB using receptor-mediated transcytosis (RMT) strategies.

The primary barrier in the lungs that siRNAs have to pass is the extracellular barrier^48^ formed by anatomical, physiological, and metabolic features of the lungs. Extracellular barriers include the reticulate pulmonary architecture from the trachea to the alveoli (Fig. 3a). The active clearance processes in this area, such as mucociliary clearance, cough clearance, and effective immune responses, inhibit the invasion of foreign material into the lungs. In addition, the presence of respiratory mucus in the upper airways and the airway surface liquid (surfactant) in the lower airways act as major physical and chemical barriers, reducing the drug penetration and diffusion rate^48^. To avoid the extracellular barrier, the proper size and density of injected particles are essential. As a drug delivery strategy, aerosolized particles are usually delivered to the lungs by inhalation. In this case, the distance at which the drug is deposited depends on the size and density of the particles. When the aerodynamic diameter is >10 µm, the drug will deposit in the nasal cavity and pharynx; particles between 2 and 10 µm will deposit in the tracheobronchial region; and finally, particles between 0.5 to 1 μm will deposit on lower bronchioles and alveoli^49^. Therefore, it is necessary to use the optimal scale for the pulmonary route. Because it is known that viral vectors increase cell uptake and siRNA efficacy, attempts have been made to overcome barriers using them. However, applying viral vectors to human therapeutics presents problems in terms of uncontrolled viral replication, immunogenicity, tumorigenicity, and toxicity^50,51^. For these reasons, the size aspect is met by utilizing naked siRNA aerosol delivery or nanoparticles^52,53^, which are nonviral vectors. Naked siRNA generally fails due to the lack of a significant gene silencing effect, but surprisingly, local delivery in the lung has shown success^54–56^.

Regarding strategies for delivering siRNAs to the lungs, it is difficult to apply animal studies directly to human studies^57^ because of the anatomical differences in the lungs between animals and humans^58^. The numbers of lobes on each side of the lungs and patterns of airway branching differ between humans and rodents. In addition, human lungs contain small intrasegmental bronchi and respiratory bronchioles that are absent or rare in rodents. Thus, the administration routes used in animal studies are unsuitable for humans, and it is difficult to measure the efficiency before starting clinical research. Even if an animal study is conducted, human lung delivery must be considered.

Among many siRNA studies to target the pulmonary system, there are two candidates that have reached the clinical stage: ALN-RSV01 and MIR 19 (siR-7-EM/KK-46) (Table 3). ALN-RSV01, developed to be administered by nasal spray as an antiviral drug, silences the nucleocapsid protein transcripts of respiratory syncytial virus (RSV). It was confirmed that RSV infection was reduced in a phase 2 clinical trial. However, there was no further progress in phase 2a and 2b trials, and the clinical trial could not proceed to the end. MIR 19 was developed with inhalation administration as a COVID-19 treatment. MIR 19 inhibits viral replication by targeting SARS-CoV-2 RNA-dependent RNA polymerase (RdRp). It has progressed to the completion of a phase 2 clinical trial.

**Table 3:** List of siRNAs in phase 2 and 3 clinical trials targeting the challenging organs: lungs, kidneys, and brain.

Target organ	Drug name	Delivery system	Target gene	Disease	Status
**Lung**	ALN-RSV01	naked siRNA	RSV nucleocapsid messenger RNA	RSV01	Phase 2, completed, NCT00658086 discontinued
	MIR 19(siR-7-EM/KK-46)	Nanoparticle	RdRp	COVID-19	Phase 2, completed, NCT05184127
**Kidney**	Teprasiran (I5NP, QPI-1002)	naked siRNA	P53	AKI	Phase 3, completed NCT02610296

*Resource:* http://clinicaltrials.gov.

### Kidneys

The kidneys have an important role in filtering blood and eliminating wastes generated in the body. They also play a homeostatic role by regulating electrolytes and water to maintain the acid-base balance and blood pressure^59^. The diverse cell types^60^ and structural complexity^61^ are the major barriers for siRNA drugs to target the kidneys. While the liver and ocular tissue consist of 4 and 5 cell types, respectively, the kidneys consist of at least 26 cell types^60^. These diverse cell types of the kidneys make it difficult to optimize and deliver drugs to specific cell types.

The glomerulus, one of the major constituents of nephrons, has a glomerular filtration barrier that acts as a barrier to the delivery of siRNA drugs (Fig. 3b). The glomerular filtration barrier comprises the endothelial fenestration, glomerular basement membrane, and podocyte extension filtration slits that are 70–90, 2–8, and 4–11 nm in diameter, respectively. Therefore, the size of the drug is critical for crossing this glomerular filtration barrier. Only small molecules with diameters less than 6 nm can pass through the glomerular filtration barrier^62^. Glomerular mesangial cells are the major targets of siRNA delivery in the glomerulus^63–65^. For targeting mesangial cells, siRNA drugs should be larger than 6 nm to prevent filtering by the urinary tract but smaller than 70–90 nm to be captured in the glomerulus and pass through the endothelial fenestration^66^. Polyethylene glycol (PEG)-poly L-lysine (PLL) nanocarriers^65^ and LNPs^64^ were used as vehicles for siRNA delivery. siRNAs in PEG-PLL carriers and LNPs have been delivered to the glomerulus via intraperitoneal^65^ and intravenous injections^64^. Naked siRNAs through the renal artery have also been tried to target mesangial cells^63^.

The tubular system, the other part of the nephrons, reabsorbs endogenous compounds. Most RNA-based studies have attempted to treat kidney disease by targeting proximal tubular cells^67–69^. Proximal tubular cells can be targeted from either the apical side (facing the tubular lumen) or the basolateral side (facing the interstitium). Only naked siRNA, which is 3–6 nm in size, can be applied from the apical side because siRNA drugs need to pass through the glomerular filtration barrier (6–7 nm) and be reabsorbed into the proximal tubular cells. Particles of siRNA carriers with larger sizes can access tubular cells from the basolateral side. They enter the tubulointerstitium by capillary pressure and are absorbed into proximal epithelial cells^70^. Many different strategies for delivering siRNAs to tubular cells have been investigated^71–73^. If oligonucleotides are accommodated in nanoparticles, they can pass through the glomerular filtration barrier only in the condition of glomerular injury. This has led to extensive studies on developing various strategies for delivering siRNAs to tubular cells^71–74^. However, direct injection into the kidneys, such as intraparenchymal injection^65^, retrograde ureter injection^67^, renal vein injection^71^, and renal artery injection^73^, has been mostly used in many preclinical studies because it can avoid the size restriction caused by the glomerular filtration barrier. The direct injection method also has the advantage of being able to target the kidney locally, avoiding accumulation in the liver. However, it has not been well applied to humans because the method is too invasive and difficult to administer^75^.

Among many studies on siRNA targeting the kidneys, one candidate has reached the clinical stage (Table 3). Teprasiran (I5NP, QPI-1002), a naked p53 siRNA, was investigated to treat acute kidney injury delayed graft function (DGF) after transplantation and cardiac surgery. Teprasiran reduces the expression of the proapoptotic protein P53 to protect the kidneys from cell death resulting from acute ischemia‒reperfusion injury and to maintain tissue and organ integrity^76^. Additionally, it was designated an orphan drug because of its efficacy in a phase 3 pivotal trial for DGF.

### Brain

The brain is an exceptional and extremely friable organ in the human body. Because the CNS is an essential system that monitors and coordinates the functions of internal organs and responds to changes in the environment, it must be protected from both endogenous and exogenous threats.

The biggest obstacle in targeting the brain with siRNA is the blood‒brain barrier (BBB) (Fig. 3c). The BBB separates the cerebrospinal fluid and blood, protecting the brain from pathogens such as viruses and various harmful substances, and selectively controls the movement of ions and molecules to regulate brain homeostasis. Brain capillary endothelial cells (BCECs), known as the thin layer of the BBB, have tight junctions (TJs) for molecules that strongly inhibit the passage of hydrophilic substances over 300 Da between cells, which is called a “physical barrier function.” Therefore, almost 98% of molecules cannot pass through the BBB, with the sole exception of lipid-soluble small molecules with a molecular weight <400 Da^77^. Because siRNAs are hydrophilic and highly negatively charged molecules with a molecular weight of ~14 kDa, it is challenging for them to pass through the BBB^78,79^.

Even if siRNAs pass through the BBB and reach the brain, the endocytosis efficiency of highly negatively charged siRNAs is very low^80^. In addition, the drug cannot be controlled to target specific areas of the brain or specific cell types in the brain. The durability of siRNAs in the brain is another issue. When siRNAs were injected into the brain parenchyma, the silencing effect was observed only in cells close to the injection site for a short time^81^ Increasing the siRNA dose may provide sufficient effects of target gene suppression in the brain. However, a high dose of siRNAs can cause strong side effects in nontargeted cells in the brain^82^. Recent studies have reported that long-lasting siRNAs are expressed in a broad area of the brain when delivered into the brain through the cerebrospinal fluid (CSF)^83,84^. A divalent siRNA (di-siRNA), in which two siRNAs are chemically conjugated, contains enough phosphorothioates in its backbone to help cellular uptake and promote a broad distribution^83^. siRNA conjugated with 2′-O-hexadecyl (C16) is broadly distributed and efficiently suppresses a target gene for a long time^84^.

Although injection methods have been utilized in preclinical research to deliver siRNAs to the brain, nanoparticles (NPs) have been used in clinical trials to deliver siRNAs to the brain. Receptor-mediated transcytosis (RMT), cell-mediated transport, carrier-mediated transport, adsorptive-mediated transcytosis, and a method for breaking the integrity of tight junctions are being used as strategies to allow nanoparticles to pass through the BBB^85,86^ (Fig. 3c). Most of the siRNA nanoparticles shown to cross the BBB use the RMT strategy, which is known to transport a wide range of proteins using the vesicular trafficking machinery in brain endothelial cells. Among them, transferrin (Tf) and rabies viral glycoprotein (RVG) tags are the most widely used^87^. Tf and RVG bind to the transferrin receptor (TfR) and nicotinic acetylcholine receptor (nAchR), respectively, both of which are located in the endothelial cells of the brain^88,89^. TfR is widely expressed throughout the human body, including the brain, but because nAchR is expressed only in the brain, brain-specific targeting is possible. This RVG strategy has been applied to suppress HMGB1, mHTT, and BACE1, which are genes related to ischemic stroke, Huntington’s disease, and Alzheimer’s disease, respectively^90–92^. In addition to RVG and Tf, apolipoprotein E3-reconstituted high-density lipoprotein (ApoE-rHDL)^93^, angiopep-2^94^, leptin^95^, and T7 peptides^96^ are also promising candidates for RMT.

Delivery systems for siRNA targeting the brain have been continually developed. One drug, NU-0129, has progressed to a clinical trial. This drug has not yet entered phase 2, but it is the only brain-targeting siRNA that has progressed to a clinical trial. NU-0129 is a spherical nucleic acid (SNA) siRNA that targets the glioblastoma oncogene BCL2L12 and crosses the BBB using the RMT strategy. SNA consists of nanoparticles in which the siRNA duplex is densely bound to the spherical gold surface. BCL2L12 is overexpressed in glioblastoma, inhibiting apoptosis and P53, thereby promoting cancer growth. An early phase 1 study was conducted in 2020, and its safety profile was confirmed^97^.

## Conclusion

RNA therapy is a promising treatment for a wide range of diseases, including cancer, cardiovascular diseases, neurodegenerative diseases, inflammatory conditions, viral infections, and eye diseases. siRNAs have the advantages of higher specificity than chemical drugs and a high degree of safety. siRNAs are also good in terms of efficiency, and candidate groups of siRNA drugs can be developed quickly and easily. All siRNA drugs approved as of 2022 target the liver, but understanding and researching siRNAs and their delivery to diverse organs besides the liver continue to be a goal. The liver has the hepatocyte-specific receptor ASGPR to which GalNAc-conjugated siRNAs can bind with high affinity. In addition, the eye and skin have a structure with good accessibility for drugs. The lungs, kidneys, and brain have complex structures that limit drug size, and the brain, in particular, has the most exceptional barrier, the BBB. Nevertheless, to overcome these barriers, siRNA drugs using various RNA modifications, conjugation systems, and delivery systems are being tested in the preclinical and clinical stages. Through understanding and researching each organ in terms of siRNA delivery, siRNA drugs targeting other organs beyond the liver are expected to emerge.

## References

[1] Izant JG, Weintraub H (1984). Inhibition of thymidine kinase gene expression by anti-sense RNA: a molecular approach to genetic analysis. Cell.

[2] Harland R, Weintraub H (1985). Translation of mRNA injected into Xenopus oocytes is specifically inhibited by antisense RNA. J. Cell Biol.

[3] Melton DA (1985). Injected anti-sense RNAs specifically block messenger RNA translation in vivo. Proc. Natl Acad. Sci. USA.

[4] Fire A (1998). Potent and specific genetic interference by double-stranded RNA in *Caenorhabditis elegans*. Nature.

[5] Elbashir SM (2001). Duplexes of 21-nucleotide RNAs mediate RNA interference in cultured mammalian cells. Nature.

[6] Noland CL, Doudna JA (2013). Multiple sensors ensure guide strand selection in human RNAi pathways. RNA.

[7] Rand TA, Petersen S, Du F, Wang X (2005). Argonaute2 cleaves the anti-guide strand of siRNA during RISC activation. Cell.

[8] Jackson AL, Linsley PS (2010). Recognizing and avoiding siRNA off-target effects for target identification and therapeutic application. Nat. Rev. Drug Discov..

[9] Qiu S, Adema CM, Lane T (2005). A computational study of off-target effects of RNA interference. Nucleic Acids Res..

[10] De Vivo M, Dal Peraro M, Klein ML (2008). Phosphodiester cleavage in ribonuclease H occurs via an associative two-metal-aided catalytic mechanism. J. Am. Chem. Soc..

[11] Schlee M, Hornung V, Hartmann G (2006). siRNA and isRNA: two edges of one sword. Mol. Ther..

[12] Garba AO, Mousa SA (2010). Bevasiranib for the treatment of wet, age-related macular degeneration. Ophthalmol. Eye Dis..

[13] Kaiser PK (2010). RNAi-based treatment for neovascular age-related macular degeneration by Sirna-027. Am. J. Ophthalmol..

[14] Puthenveetil S (2006). Controlling activation of the RNA-dependent protein kinase by siRNAs using site-specific chemical modification. Nucleic Acids Res..

[15] Broering R (2014). Chemical modifications on siRNAs avoid Toll-like-receptor-mediated activation of the hepatic immune system in vivo and in vitro. Int. Immunol..

[16] Zhu Y, Zhu L, Wang X, Jin H (2022). RNA-based therapeutics: an overview and prospectus. Cell Death Dis..

[17] Dhuri K (2020). Antisense oligonucleotides: an emerging area in drug discovery and development. J. Clin. Med..

[18] Shen W (2019). Chemical modification of PS-ASO therapeutics reduces cellular protein-binding and improves the therapeutic index. Nat. Biotechnol..

[19] Crooke ST, Vickers TA, Liang X-H (2020). Phosphorothioate modified oligonucleotide–protein interactions. Nucleic Acids Res..

[20] Liang X-h (2021). Solid-phase separation of toxic phosphorothioate antisense oligonucleotide-protein nucleolar aggregates is cytoprotective. Nucleic Acid Ther..

[21] Hu B (2020). Therapeutic siRNA: state of the art. Signal Transduct. Target. Ther..

[22] Friedrich M, Aigner A (2022). Therapeutic siRNA: state-of-the-art and future perspectives. BioDrugs.

[23] Kristen AV (2019). Patisiran, an RNAi therapeutic for the treatment of hereditary transthyretin-mediated amyloidosis. Neurodegener. Dis. Manag..

[24] Scott LJ (2020). Givosiran: first approval. Drugs.

[25] Scott LJ, Keam SJ (2021). Lumasiran: first approval. Drugs.

[26] Ray KK (2020). Two phase 3 trials of inclisiran in patients with elevated LDL cholesterol. N. Engl. J. Med..

[27] Mullard A (2022). FDA approves fifth RNAi drug - Alnylam’s next-gen hATTR treatment. Nat. Rev. Drug Discov..

[28] Almazroo OA, Miah MK, Venkataramanan R (2017). Drug metabolism in the liver. Clin. Liver Dis..

[29] Holm A, Lovendorf MB, Kauppinen S (2021). Development of siRNA therapeutics for the treatment of liver diseases. Methods Mol. Biol..

[30] Hirsjarvi S, Passirani C, Benoit JP (2011). Passive and active tumour targeting with nanocarriers. Curr. Drug Discov. Technol..

[31] Tang Y (2019). Overcoming the reticuloendothelial system barrier to drug delivery with a “Don’t-Eat-Us” strategy. ACS Nano.

[32] Debacker AJ, Voutila J, Catley M, Blakey D, Habib N (2020). Delivery of oligonucleotides to the liver with GalNAc: from research to registered therapeutic drug. Mol. Ther..

[33] Streilein JW (2003). Ocular immune privilege: therapeutic opportunities from an experiment of nature. Nat. Rev. Immunol..

[34] Novack GD (2009). Ophthalmic drug delivery: development and regulatory considerations. Clin. Pharmacol. Ther..

[35] Jiang J, Zhang X, Tang Y, Li S, Chen J (2021). Progress on ocular siRNA gene-silencing therapy and drug delivery systems. Fundam. Clin. Pharmacol..

[36] Bachu RD, Chowdhury P, Al-Saedi ZHF, Karla PK, Boddu SHS (2018). Ocular drug delivery barriers-role of nanocarriers in the treatment of anterior segment ocular diseases. Pharmaceutics.

[37] Benitez-Del-Castillo JM (2016). Safety and efficacy clinical trials for SYL1001, a novel short interfering RNA for the treatment of dry eye disease. Invest. Ophthalmol. Vis. Sci..

[38] Zahir-Jouzdani F (2018). Corneal chemical burn treatment through a delivery system consisting of TGF-beta(1) siRNA: in vitro and in vivo. Drug Deliv. Transl. Res..

[39] Chong DY, Anand R, Williams PD, Qureshi JA, Callanan DG (2010). Characterization of sterile intraocular inflammatory responses after intravitreal bevacizumab injection. Retina.

[40] Shen J, Durairaj C, Lin T, Liu Y, Burke J (2014). Ocular pharmacokinetics of intravitreally administered brimonidine and dexamethasone in animal models with and without blood-retinal barrier breakdown. Invest. Ophthalmol. Vis. Sci..

[41] Jiang S, Chen X (2017). HMGB1 siRNA can reduce damage to retinal cells induced by high glucose in vitro and in vivo. Drug Des. Devel. Ther..

[42] Gilaberte, Y., Prieto-Torres, L., Pastushenko, I. & Juarranz, Á. Anatomy and Function of the Skin. *Nanoscience in Dermatology* 1–14 (2016).

[43] Prausnitz MR, Mitragotri S, Langer R (2004). Current status and future potential of transdermal drug delivery. Nat. Rev. Drug Discov..

[44] Benson HAE, Grice JE, Mohammed Y, Namjoshi S, Roberts MS (2019). Topical and transdermal drug delivery: from simple potions to smart technologies. Curr. Drug Deliv..

[45] Geusens B (2010). Flexible nanosomes (SECosomes) enable efficient siRNA delivery in cultured primary skin cells and in the viable epidermis of ex vivo human skin. Adv. Funct. Mater..

[46] Bracke S (2014). Targeted silencing of DEFB4 in a bioengineered skin-humanized mouse model for psoriasis: development of siRNA SECosome-based novel therapies. Exp. Dermatol..

[47] Colwell AS, Phan TT, Kong W, Longaker MT, Lorenz PH (2005). Hypertrophic scar fibroblasts have increased connective tissue growth factor expression after transforming growth factor-beta stimulation. Plast. Reconstr. Surg..

[48] Sanders N, Rudolph C, Braeckmans K, De Smedt SC, Demeester J (2009). Extracellular barriers in respiratory gene therapy. Adv. Drug Deliv. Rev..

[49] Thakur, A. K., Kaundle, B. & Singh, I. in *Targeting Chronic Inflammatory Lung Diseases Using Advanced Drug Delivery Systems* 475–491 (2020).

[50] Raper SE (2003). Fatal systemic inflammatory response syndrome in a ornithine transcarbamylase deficient patient following adenoviral gene transfer. Mol. Genet. Metab..

[51] Bessis N, GarciaCozar FJ, Boissier MC (2004). Immune responses to Gene Ther.apy vectors: influence on vector function and effector mechanisms. Gene Ther..

[52] Bai X (2022). Inhaled siRNA nanoparticles targeting IL11 inhibit lung fibrosis and improve pulmonary function post-bleomycin challenge. Sci. Adv..

[53] Keil TWM, Baldassi D, Merkel OM (2020). T-cell targeted pulmonary siRNA delivery for the treatment of asthma. Wiley Interdiscip. Rev. Nanomed. Nanobiotechnol..

[54] Fulton A (2009). Effective treatment of respiratory alphaherpesvirus infection using RNA interference. PLoS ONE.

[55] Li BJ (2005). Using siRNA in prophylactic and therapeutic regimens against SARS coronavirus in Rhesus macaque. Nat. Med..

[56] Bitko V, Musiyenko A, Shulyayeva O, Barik S (2005). Inhibition of respiratory viruses by nasally administered siRNA. Nat. Med..

[57] Hofmann W, Koblinger L, Martonen TB (1989). Structural differences between human and rat lungs: implications for Monte Carlo modeling of aerosol deposition. Health Phys..

[58] Meyerholz, D. K., Suarez, C. J., Dintzis, S. M. & Frevert, C. W. in *Compar. Anatom. Histol.* 147–162 (2018).

[59] Ruggiero A (2010). Paradoxical glomerular filtration of carbon nanotubes. Proc. Natl Acad. Sci. USA.

[60] Schumacher A (2021). Defining the variety of cell types in developing and adult human kidneys by single-cell RNA sequencing. NPJ Regen. Med..

[61] Jourde-Chiche N (2019). Endothelium structure and function in kidney health and disease. Nat. Rev. Nephrol..

[62] Huang J, Gretz N (2017). Light-emitting agents for noninvasive assessment of kidney function. ChemistryOpen.

[63] Takabatake Y, Isaka Y, Imai E (2009). In vivo transfer of small interfering RNA or small hairpin RNA targeting glomeruli. Methods Mol. Biol..

[64] Wang Y (2020). Co-delivery of p38alpha MAPK and p65 siRNA by novel liposomal glomerulus-targeting nano carriers for effective immunoglobulin a nephropathy treatment. J. Control. Release.

[65] Shimizu H (2010). siRNA-based therapy ameliorates glomerulonephritis. J. Am. Soc. Nephrol..

[66] Wang J, Masehi-Lano JJ, Chung EJ (2017). Peptide and antibody ligands for renal targeting: nanomedicine strategies for kidney disease. Biomater. Sci..

[67] Xia Z (2008). Suppression of renal tubulointerstitial fibrosis by small interfering RNA targeting heat shock protein 47. Am. J. Nephrol..

[68] Alidori S (2016). Targeted fibrillar nanocarbon RNAi treatment of acute kidney injury. Sci. Transl. Med..

[69] Morishita Y (2014). siRNAs targeted to Smad4 prevent renal fibrosis in vivo. Sci. Rep..

[70] Dolman ME, Harmsen S, Storm G, Hennink WE, Kok RJ (2010). Drug targeting to the kidney: advances in the active targeting of therapeutics to proximal tubular cells. Adv. Drug Deliv. Rev..

[71] Hamar P (2004). Small interfering RNA targeting Fas protects mice against renal ischemia-reperfusion injury. Proc. Natl Acad. Sci. USA.

[72] Zheng X (2016). Attenuating ischemia-reperfusion injury in kidney transplantation by perfusing donor organs with siRNA cocktail solution. Transplantation.

[73] Yang B, Hosgood SA, Nicholson ML (2011). Naked small interfering RNA of caspase-3 in preservation solution and autologous blood perfusate protects isolated ischemic porcine kidneys. Transplantation.

[74] Liu L (2012). Small interfering RNA targeting Toll-like receptor 9 protects mice against polymicrobial septic acute kidney injury. Nephron Exp. Nephrol..

[75] Bondue T, van den Heuvel L, Levtchenko E, Brock R (2022). The potential of RNA-based therapy for kidney diseases. Pediatr. Nephrol..

[76] Thompson JD (2012). Toxicological and pharmacokinetic properties of chemically modified siRNAs targeting p53 RNA following intravenous administration. Nucleic Acid Ther..

[77] Pardridge WM (2022). A historical review of brain drug delivery. Pharmaceutics.

[78] Dowdy SF (2017). Overcoming cellular barriers for RNA therapeutics. Nat. Biotechnol..

[79] Khvorova A, Osborn MF, Hassler MR (2014). Taking charge of siRNA delivery. Nat. Biotechnol..

[80] Zhang W, Mehta A, Tong Z, Esser L, Voelcker NH (2021). Development of polymeric nanoparticles for blood-brain barrier transfer-strategies and challenges. Adv. Sci. (Weinh).

[81] Gomes MJ, Martins S, Sarmento B (2015). siRNA as a tool to improve the treatment of brain diseases: mechanism, targets and delivery. Ageing Res. Rev..

[82] Murthy SK (2007). Nanoparticles in modern medicine: state of the art and future challenges. Int. J. Nanomed..

[83] Alterman JF (2019). A divalent siRNA chemical scaffold for potent and sustained modulation of gene expression throughout the central nervous system. Nat. Biotechnol..

[84] Brown KM (2022). Expanding RNAi therapeutics to extrahepatic tissues with lipophilic conjugates. Nat. Biotechnol..

[85] Chen Y, Liu L (2012). Modern methods for delivery of drugs across the blood-brain barrier. Adv. Drug Deliv. Rev..

[86] Saraiva C (2016). Nanoparticle-mediated brain drug delivery: overcoming blood-brain barrier to treat neurodegenerative diseases. J. Control. Release.

[87] Lajoie JM, Shusta EV (2015). Targeting receptor-mediated transport for delivery of biologics across the blood-brain barrier. Annu. Rev. Pharmacol. Toxicol..

[88] Clark AJ, Davis ME (2015). Increased brain uptake of targeted nanoparticles by adding an acid-cleavable linkage between transferrin and the nanoparticle core. Proc. Natl Acad. Sci. USA.

[89] Kumar P (2007). Transvascular delivery of small interfering RNA to the central nervous system. Nature.

[90] Zhang L (2021). Therapeutic reversal of Huntington’s disease by in vivo self-assembled siRNAs. Brain.

[91] Kim M, Kim G, Hwang DW, Lee M (2019). Delivery of high mobility group box-1 siRNA using brain-targeting exosomes for ischemic stroke therapy. J. Biomed. Nanotechnol..

[92] Alvarez-Erviti L (2011). Delivery of siRNA to the mouse brain by systemic injection of targeted exosomes. Nat. Biotechnol..

[93] Huang JL (2017). Lipoprotein-biomimetic nanostructure enables efficient targeting delivery of siRNA to Ras-activated glioblastoma cells via macropinocytosis. Nat. Commun..

[94] Gao S (2016). A non-viral suicide gene delivery system traversing the blood brain barrier for non-invasive glioma targeting treatment. J. Control. Release.

[95] Liu Y (2010). A leptin derived 30-amino-acid peptide modified pegylated poly-L-lysine dendrigraft for brain targeted gene delivery. Biomaterials.

[96] Wei L (2016). Brain tumor-targeted therapy by systemic delivery of siRNA with Transferrin receptor-mediated core-shell nanoparticles. Int. J. Pharm..

[97] Kumthekar P (2021). A first-in-human phase 0 clinical study of RNA interference-based spherical nucleic acids in patients with recurrent glioblastoma. Sci. Transl. Med..

